# Label-Free Quantitative Proteomics Reveals Differences in Molecular Mechanism of Atherosclerosis Related and Non-Related to Chronic Kidney Disease

**DOI:** 10.3390/ijms17050631

**Published:** 2016-05-02

**Authors:** Magdalena Luczak, Joanna Suszynska-Zajczyk, Lukasz Marczak, Dorota Formanowicz, Elzbieta Pawliczak, Maria Wanic-Kossowska, Maciej Stobiecki

**Affiliations:** 1 European Centre for Bioinformatics and Genomics, Institute of Bioorganic Chemistry, Polish Academy of Sciences, Noskowskiego 12/14, 61-704 Poznan, Poland; lukasmar@ibch.poznan.pl (L.M.); mackis@ibch.poznan.pl (M.S.); 2 Institute of Chemical Technology and Engineering, Poznan University of Technology, Piotrowo 3, 60-965 Poznan, Poland; 3 Department of Biochemistry and Biotechnology, Poznan University of Life Sciences, Dojazd 11, 60-632 Poznan, Poland; suszynska@yahoo.co.uk; 4 Department of Clinical Biochemistry and Laboratory Medicine, Poznan University of Medical Sciences, Grunwaldzka 6, 60-780 Poznan, Poland; doforman@ump.edu.pl; 5 Department of Nephrology, Transplantology and Internal Medicine, Poznan University of Medical Sciences, Przybyszewskiego 49, 60-355 Poznan, Poland; epawliczak@wp.pl (E.P.); wanic.kossowska.maria@gmail.com (M.W.-K.)

**Keywords:** chronic kidney disease, cardiovascular disease, atherosclerosis, label-free quantitative proteomics

## Abstract

The major cause of mortality in patients with chronic kidney disease (CKD) is atherosclerosis related to traditional and non-traditional risk factors. However, the understanding of the molecular specificity that distinguishes the risk factors for classical cardiovascular disease (CVD) and CKD-related atherosclerosis (CKD-A) is far from complete. In this study we investigated the disease-related differences in the proteomes of patients with atherosclerosis related and non-related to CKD. Plasma collected from patients in various stages of CKD, CVD patients without symptoms of kidney dysfunction, and healthy volunteers (HVs), were analyzed by a coupled label-free and mass spectrometry approach. Dysregulated proteins were confirmed by an enzyme-linked immunosorbent assay (ELISA). All proteomic data were correlated with kidney disease development and were subjected to bioinformatics analysis. One hundred sixty-two differentially expressed proteins were identified. By directly comparing the plasma proteomes from HVs, CKD, and CVD patients in one study, we demonstrated that proteins involved in inflammation, blood coagulation, oxidative stress, vascular damage, and calcification process exhibited greater alterations in patients with atherosclerosis related with CKD. These data indicate that the above nontraditional risk factors are strongly specific for CKD-A and appear to be less essential for the development of “classical” CVD.

## 1. Introduction

Chronic kidney disease (CKD) is highly prevalent worldwide and is an important cause of morbidity, especially due to cardiovascular disease (CVD). In classical CVD, without renal dysfunction, most atherosclerosis is caused by traditional risk factors that can be controlled, treated or modified (such as hypertension, tobacco use, diabetes, lipid levels) or factors that cannot be changed (such as age, gender, and family history) [1]. In these cases, atherosclerosis is the consequence of many years of exposure to atherogenic influences that lead to early lesions. Then, under the influence of age and different factors, such as lipid metabolism, blood pressure, and diet, these early lesions advance, and atherogenesis is accelerated. Chronic kidney disease-related atherosclerosis (CKD-A) is more complex and is related to traditional and non-traditional risk factors, including inflammation, endothelial dysfunction, oxidative stress, vascular calcification, and volume overload. All of these problems lead to hypertension, anemia, mineral and bone disorders, and vascular remodeling and damage [2]. However, these risk factors and complications, especially inflammation and endothelial dysfunction, are also related to the pathogenesis of non-renal atherosclerotic CVDs [3]. Therefore, we sought to determine which of these risk factors have a substantial role in and are specific for CKD-A.

The diagnosis of CKD is mainly based on the measured or estimated glomerular filtration rate (eGFR) and/or evidence of kidney damage (usually indicated by albuminuria or proteinuria) for a period of at least three months. The first and second stages of CKD (CKD1, CKD2) are the mildest ones, and although eGFRs are normal (above 90 mL/min/1.73 m^2^) or slightly decreased (at most 60 mL/min/1.73 m^2^), they have other evidence of renal disease like proteinuria or structural or functional abnormalities of the kidney. In the third and fourth stages of CKD, moderate and severe reduction in the eGFR are observed (CKD3: eGFR 30–59 mL/min/1.73 m^2^; CKD4: 15–29 mL/min/1.73 m^2^). The fifth stage of CKD (CKD5) is the most advanced stage and is related to kidney failure (GFR < 15 mL/min/1.73 m^2^). The latter end-stage renal disease (ESRD) patients receive renal replacement therapy. The typical eGFR in adults aged 60 years is between 60 and 90 mL/min per 1.73 m^2^. The eGFR declines during the progression of CKD and the aging process. However, among CKD patients, even a mild or moderate eGFR decrease increases the risk of serious cardiovascular events and CVD-related mortality [4].

Herein, we applied a label-free proteomic approach to screen changes in protein expression in three stages of CKD and one stage of CVD as well as in healthy volunteers (HVs) to better understand the role of individual processes, pathways, and risk factors in CKD-A. All of the patients varied in both the progression of atherosclerosis and the advancement of renal disease. Furthermore, we reported the relative quantification information for 162 differentially expressed proteins. In this study, we focused on the relationship between the altered accumulation of differential proteins and the progression of CKD-A. The obtained data were evaluated by functional annotation and validated with an enzyme-linked immunosorbent assay (ELISA).

## 2. Results

The plasma collected from five experimental groups, HV, CKD1-2, CKD3-4, CKD5, and CVD, without any fractionation were digested in solution with trypsin and analyzed by nano LC-MS/MS in one batch. Each of the 150 plasma samples was prepared for digestion in duplicates, and then, every prepared sample was injected into the LC system in duplicate at random. As a result, the 600 LC-MS/MS runs were prepared within three months without any break. Every 73 h, a calibration of the system was performed. Due to the long analysis time, it was important to ensure that there was good agreement between all of the datasets. The reproducibility of the technical and biological replicates was assessed by scatter plotting and the correlation coefficient determined based on the LFQ (label-free quantification) intensities. The Pearson correlations within each experimental group are presented in Table 1. The correlation analysis of the LFQ signal intensities between the technical and biological replications calculated for the experimental groups revealed Pearson coefficients between 0.81 and 0.99 (Table 1). These data indicated that the sample replicates had a high degree of reproducibility. The Proteome Discoverer (PD) analysis showed that the percentage overlap between the duplicate injections was higher than 90% at the protein level. The percentage overlap between the biological replicates from the same experimental group was 82% at the protein level.

### 2.1. Quantitative Analysis of Plasma Proteins

**Figure 1:**
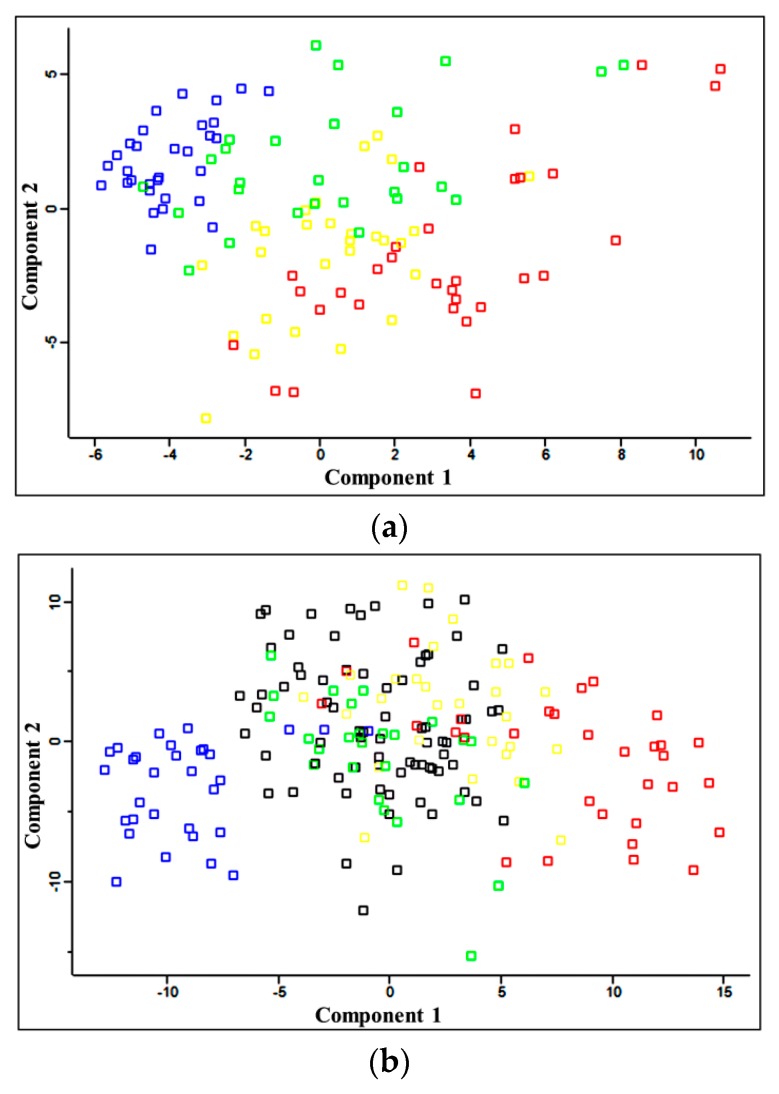
(**a**) Principal component analysis (PCA) of the LFQ intensities obtained from the plasma of HVs (**blue**), CKD1-2 (**green**), CKD3-4 (**yellow**) and CKD5 (**red**) patients; (**b**) PCA of the LFQ intensities for the HVs and all CKD as well as CVD (**black**) patients. Calculations were performed with Perseus.

The main objective of this study was the comparative proteomic analysis and identification of differentially accumulated proteins in the plasma of patients with different stages of CKD, patients with CVD (and normal renal function), and HVs to find proteins that correlated with CKD-A progression. Comparative analyses were performed between HVs and each of the groups of CKD patients, between CKD and CVD patients, and between neighboring groups of CKD patients. In this analysis, 1798 proteins were identified with one unique peptide using PD; 611 and 519 proteins were identified with a minimum of two peptides and 1% false discovery rate (FDR) using the MaxQuant (MQ) software and the PD software, respectively. Principal component analysis (PCA) differentiated the HVs and CKD1-2, CKD3-4, and CKD5 patients (Figure 1a) and revealed similarity between the CVD and CKD1-2 samples (Figure 1b).

Quantitative analysis completed by MaxQuant identified 162 proteins with a minimum of two peptides, a threshold greater than 1.5, and *p* values below 0.05 (ANOVA) and differentiated all analyzed groups of patients. All identified differential proteins are listed in Table S1. The analysis of the differentially expressed proteins revealed that 49 of them distinguished the HVs and the CKD1-2 group, and 61 and 71 proteins distinguished HVs from the CKD3-4 group and HVs from the CKD5 group, respectively. A comparison of the HVs and CVD patients revealed 42 differentially expressed proteins. ELISA validation experiments were performed to confirm the up- or down-regulation of the proteins identified in label-free analysis. The results obtained in LC-MS/MS analysis for α-1microglobulin (α1m) and β-2-microglobulin (β2m), apolipoprotein AIV (apoAIV), fibrinogen α, β, and γ, and cystatin C (cysC) were consistent with the ELISA data.

### 2.2. Pathways and Functional Annotations of Differential Proteins

**Figure 2:**
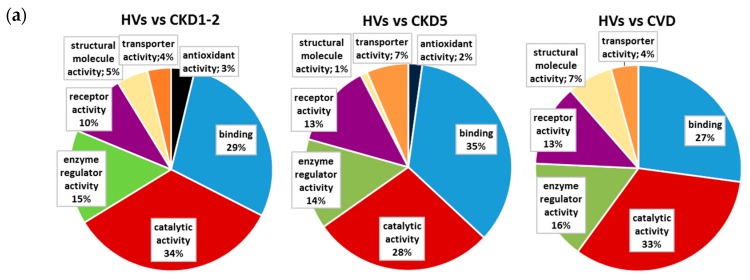

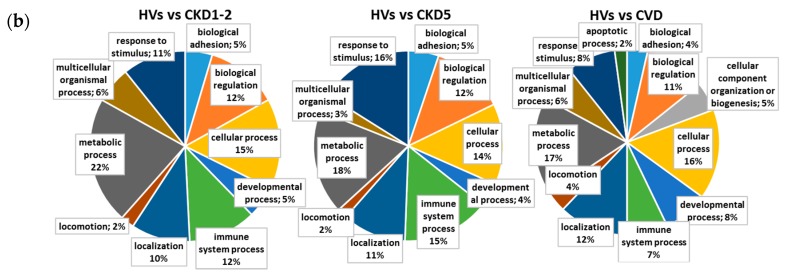
Classification of the identified differentially expressed proteins in molecular function (**a**) and biological processes; (**b**) on the basis of gene ontology (GO) annotations with *p* < 0.05.

We used two analysis tools, DAVID (Database for Annotation, Visualization, and Integrated Discovery) and PANTHER (Protein ANalysis THrough Evolutionary Relationships), to find enriched annotations in the 162 differentially expressed proteins. Proteins distinguishing HVs and particular CKD groups as well as the HVs and CVD patients were analyzed separately. The data were classified based on their respective molecular function, biological processes and physiological pathways. Our analysis showed that the proteins differentially expressed in HVs and CKD patients were classified into seven GO (gene ontology) classes of molecular function: antioxidant activity (inhibition of the reactions brought about by dioxygen or peroxides), catalytic activity (catalysis of a biochemical reaction at physiological temperature), enzyme regulator activity (binding to and modulation of the activity of an enzyme), receptor activity (acting in conjunction with an extracellular or intracellular messenger to initiate a change in cell activity), structural molecule activity (contributing to the structural integrity of a complex or assembly within or outside a cell), transporter activity (enabling the directed movement of molecules and ions into, out of, or within a cell or between cells), and binding (selective, non-covalent, and often stoichiometric interaction of a molecule with one or more specific sites on another molecule). The forty-two differentially expressed proteins in the HV/CVD comparison include all of above molecular functions, except antioxidant activity. Some of results of these analyses are presented in Figure 2a. The GO analysis of the biological processes showed the same ten GO classes in the comparison between HVs and all of the CKD patients (Figure 2b). However, the participation of particular proteins was different: 11% of the identified differential proteins were involved in response to stimulus in the HVs compared with the CKD1-2 group, whereas 14% and 16% of the proteins were involved in response to stimulus in the HVs compared with the CKD3-4 group and CKD5 group, respectively. Furthermore, a number of proteins involved in the immune system process increased according to CKD progression (12% (in HVs *vs.* the CKD1-2 group) and 15% (in HV *vs.* the CKD5 group)). The comparison between the HVs and the CVD patients revealed two additional GO classes: apoptotic processes and cellular component organization or biogenesis. However, only 8% of the differentially expressed proteins were involved in responses to stimuli, and 7% were associated with immune system processes.

The analysis of the physiological pathways revealed that the most overrepresented were hemostasis, complement cascade, inflammation mediated by the chemokine and cytokine signaling pathway, integrin cell surface interaction, signaling in the immune system, plasminogen activation cascade, cardiac muscle contraction, cardiomyopathy, and metabolism of lipids and lipoproteins (Table 2). However, the levels of the differentially expressed proteins related to blood coagulation or inflammation were twice as high in the HVs *vs.* CKD5 compared to HVs *vs.* CVD group. Proteins related to the plasminogen activation cascade, signaling in the immune system, and integrin cell surface interactions were identified only in the HV/CKD comparison. In contrast, cardiac muscle contraction and cardiomyopathy proteins were characteristic only for the HV/CVD comparison. The same number of proteins involved in the metabolism of lipids and lipoproteins differed in groups HV/CKD1-2 and HV/CVD. The number of the latter proteins was at least two times lower in the comparison between HVs and CKD5 group.

### 2.3. Proteins Specifically Related to CKD Progression

**Figure 3:**
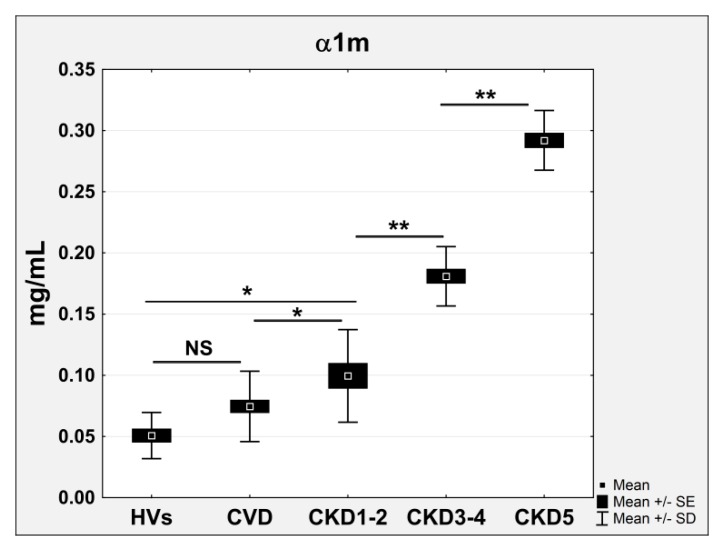
ELISA measurements of α1m. Chart shows mean, standard error (SE), and standard deviation (SD) for all analyzed plasma samples. Student’s *t*-tests were completed and statistical significance is indicated (* *p* < 0.05, ** *p* < 0.001, NS: non-significant).

The general influence of CKD progression on the plasma proteome was analyzed. The correlation between kidney disease development and the relative accumulation of identified differential proteins was calculated on the basis of the eGFR of all analyzed patients and the LFQ intensities determined for differential proteins. Proteins with correlation coefficients above 0.65 and below −0.65 were considered to be CKD-related. The levels of 29 proteins were associated with CKD progression and consequently with the eGFR level (Table 3). Twenty-three of these proteins were negatively correlated with eGFR (correlation coefficients between −0.82 and −0.68), and the levels of six proteins were positively correlated and decreased along with eGFR decline (correlation coefficients between 0.71 and 0.77). Among these proteins, the accumulation levels of β2m and α1m were increased in the plasma of CKD and CVD patients together with decreasing eGFR levels (*r* = −0.79). The accumulation of β2m and α1m was 1.52 and 1.4 times higher in CVD patients, 2.46 and 2.04 times higher in CKD1-2 patients, 8.09 and 2.86 higher in the CKD3-4 group, and 32.04 and 4.89 times higher in CKD5 patients, respectively. The altered abundance of β2m and α1m was confirmed by the ELISA results, and the result for α1m is presented on Figure 3.

**Figure 4:**
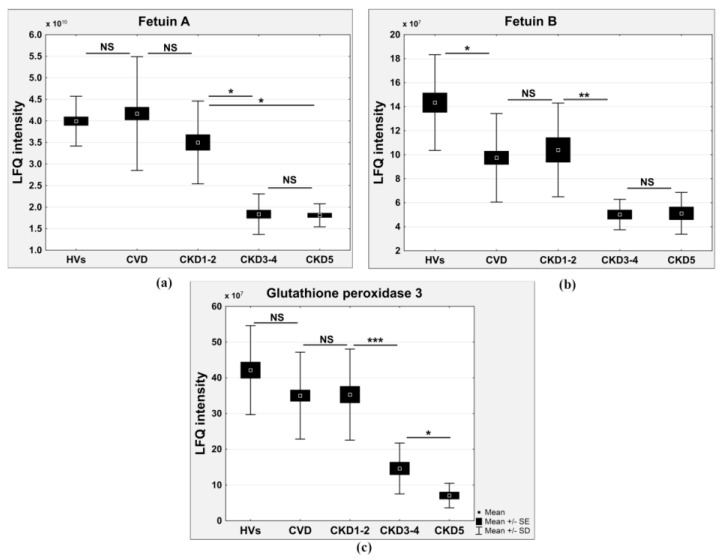
Relative abundance of fetuin A (**a**) fetuin B; (**b**) and glutathione peroxidase 3; (**c**) in HVs, CVD, CKD1-2, CKD3-4, and CKD5 groups. Student’s *t*-tests were completed and statistical significance is indicated (* *p* < 0.05, ** *p* < 0.001, *** *p* < 0.0001, NS: non-significant).

Compared to the HVs, the relative abundances of fetuin A, fetuin B, and glutathione peroxidase 3 were decreased along with the decreases in eGFR (*r* = 0.73, 0.77, and 0.76; fold changes of 0.46, 0.4, and 0.16 in the CKD5/HV comparison and 0.5, 0.35, and 0.33 in CKD3-4/HV comparison for fetuin A, fetuin B, and glutathione peroxidase 3, respectively) (Figure 4a–c).

**Figure 5:**
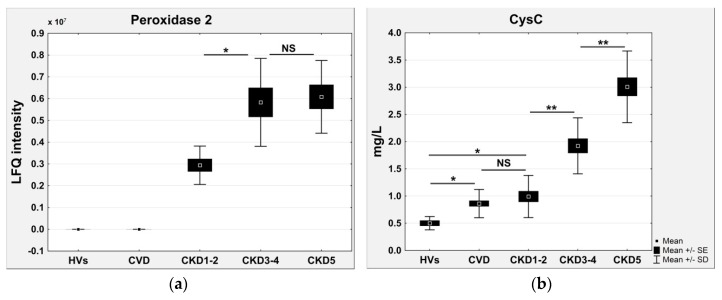
(**a**) Relative abundance of peroxiredoxin 2 (PRDX2) in HVs, CVD, CKD1-2, CKD3-4, and CKD5 groups based on LFQ intensities; (**b**) ELISA measurements of cysC. Charts show mean, SE, and SD for all analyzed plasma samples. Student’s *t*-tests were completed and statistical significance is indicated (* *p* < 0.05, ** *p* < 0.001, NS: non-significant).

A second set of identified differential proteins consisted of proteins that were completely undetectable in the HVs (Table 3). The accumulation of these proteins was observed in all CKD and CVD samples and was proportional to the eGFR decline. For example, the relative amounts of peroxiredoxin 2 (PRDX2) (Figure 5a) and cysC were increased in the plasma of CVD and CKD patients, together with a decline in the eGFR. The accumulation of PRDX2 and cysC was 2.03 and 4.25 times higher in the plasma of CKD3-4 patients and 2.06 and 6.32 times higher in the plasma of CKD5 patients compared to the CKD1-2 group, respectively. The correlation coefficients for these proteins are presented in Table 3. The altered abundance of cysC was confirmed by the ELISA results (Figure 3b). The concentration of this protein in the ELISA test in HVs was only 0.56 ± 0.12 mg/L, which may explain why no signal was observed in the LC/MS/MS analysis.

**Figure 6:**
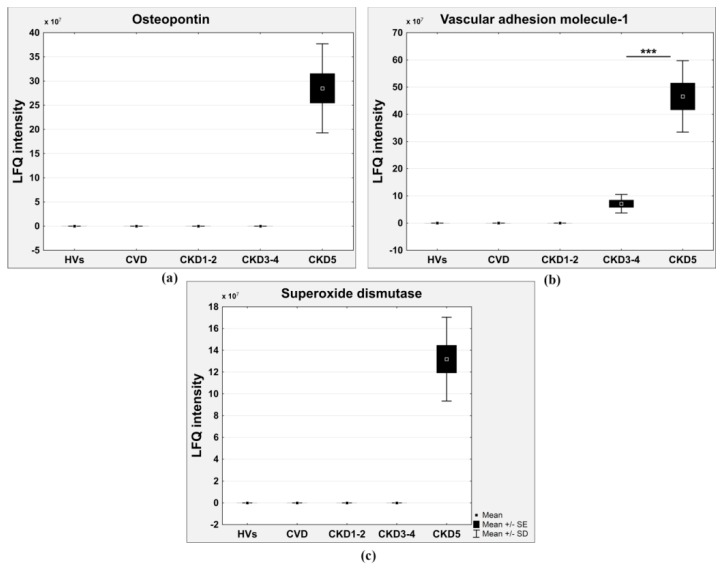
Relative abundance of osteopontin (**a**) VCAM1; (**b**) and superoxide dismutase; (**c**) in HVs, CKD1-2, CKD3-4, CKD5, and CVD groups. (*** *p* < 0.0001).

A third set of differential proteins was also absent in the CVD and CKD1-2 groups and even in the CKD3-4 group (Table 2). For example, the accumulation of osteopontin (Figure 6a), uteroglobin, calreticulin, CD59 glycoprotein, vascular adhesion molecule-1 (VCAM1) (Figure 6b), prostaglandin-H2 D-isomerase, superoxide dismutase (Figure 6c), and guanylin was detected only in the most advanced CKD stages.

All of the data presented in Table 3 were considered to be proteins characteristic of CKD and related to eGFR decline. Thus, the alteration levels of these proteins were proportional to the level of CKD progression. A correlation analysis was also performed for the obtained immunoassay results and revealed a negative correlation between eGFR and the concentrations of cysC, α1m, β2m, and fibrinogen α, β, and γ (*r* = −0.850, *p* = 0.002, *r* = −0.778, *p* = 0.001, *r* = −0.796, *p* = 0.001, *r* = −0.7502, *p* = 0.005, *r* = −0.7401, *p* = 0.005, and *r* = −0.680, *p* = 0.002, respectively).

In the last step, a molecular function and pathway analysis was performed only for proteins related to CKD. The obtained results showed that most proteins related to CKD were associated with blood coagulation and the hemostasis pathway (with Benjamini-corrected *p* values of 9.6 × 10^−6^ and 5.7 × 10^−12^, respectively). Other proteins were classified into the following pathways and GO classes: signaling in the immune system, inflammation mediated by the chemokine and cytokine signaling pathway, cytokine secretion and the inflammatory response, (*p* = 6.4 × 10^−5^, 7.8 × 10^−6^ and 2.4 × 10^−6^, respectively), calcium metabolism and calcium ion binding (*p* = 1.8 × 10^−5^ and 1.4 × 10^−6^, respectively), and the cellular response to oxidative stress/detoxification of reactive oxygen species (*p* = 3.2 × 10^−2^ and 2.8 × 10^−2^). The relative abundance of all proteins related to CKD progression confirmed the results obtained in the PCA. The PCA differentiated the HVs, CKD1-2, CKD3-4, and CKD5 groups (Figure 1a) and revealed their similarities, especially between the CVD and CKD1-2 samples (Figure 1b), even though patients from the CVD and CKD1-2 groups differed considerably in the history of their cardiac events and the progress of atherosclerosis. Twenty identified proteins had similar accumulation in the CVD and CKD1-2 patients but were completely different in the CVD and CKD5 groups (Table S2). Obtained results revealed that with the degree of CKD progression increases the number of proteins with diverse accumulation that are involved in the response to external stimuli and systemic inflammatory processes, plasma acute phase proteins, calcium metabolism proteins, and molecules related to oxidative stress. In patients with “classical” CVD, we also observed a diverse accumulation of certain inflammatory proteins, but they were fewer in number (three times fewer proteins than in patients with the most advanced CKD). Furthermore, the changes were lower in magnitude than in the patients with CKD. This finding was also confirmed by the measured level of serum C-reactive protein (CRP), a marker of systemic inflammation. The level of this protein was 1.09 ± 0.15 mg/L in the HV group, 1.62 ± 0.36 mg/L in the CKD1-2 group, 9.19 ± 2.15 mg/L in the CKD3-4 group, and 12.32 ± 18.05 mg/L in the CKD5 group, whereas in the CVD patients, the CRP level was 5.88 ± 1.14 mg/L.

## 3. Discussion

Several methods for relative proteomic quantitation have been described in recent years [5,6]. Labeling methods, including iTRAQ analysis, can be expensive but generally require fewer LC-MS/MS runs to generate robust results [7]. In label-free quantitation strategies, each sample must be analyzed separately with replicates for a high level of reproducibility; however, this technique is extremely convenient due to the simplicity of sample preparation. Moreover, the capabilities of the Q-Exactive Orbitrap spectrometer make a label-free approach very attractive and precise in quantitative protein analysis. In combination with the MaxQuant software, Q-Exactive enabled the identification of 611 plasma proteins with two or more unique peptides with 99% confidence. Additionally, the obtained results revealed a high level of run-to-run and sample-to-sample reproducibility, confirming that two experimental and two injection replicates were sufficient for the precise determination of protein ratios in label-free studies of plasma. The high level of run-to-run and sample-to-sample reproducibility was probably obtained because the plasma protein procedure did not require complex and multistep methods for isolation and purification. We have previously shown that one of the main drawbacks of removing abundant proteins from plasma using an affinity column is the simultaneous removal of non-targeted proteins [8]. We have also shown that strong cation exchange (SCX) chromatography without affinity depletion is a suitable plasma sample pretreatment method for proteomic analysis. However, the preparation of 150 samples in this manner would be extremely time consuming. Instead, we decided to use a simple method of plasma protein sample preparation and extend the LC separation to 230 min. In this way we obtained very reliable data. The results of the PCA analysis by Perseus are shown in Figure 1. PCA allowed us to separate all of the analyzed CKD experimental groups according to protein abundance variation, which was helpful in interpreting the relationships between the experimental groups. The pathway analysis showed that most of the differentially expressed proteins related to CKD progression were linked with hemostasis, inflammation and the inflammatory response, calcium ion metabolism, and the cellular response to oxidative stress.

Both conditions, CKD and CVD, are chronic inflammatory diseases [9,10]. Chronic inflammation and endothelial dysfunction, resulting in the disintegration of vascular structure and its function, are key elements in the progression of both atherosclerosis and kidney failure. CKD patients, especially ESRD patients treated with hemodialysis, are exposed to vessel damage during each dialysis session because of the contact of blood with the dialysis membrane [11,12]. Our study confirmed that inflammation is more pronounced in CKD patients than in CVD patients. This finding is supported by the differential accumulation of proteins that are involved in immune reactions and act as acute phase proteins. Among proteins associated with CKD progression β2m, α1m, two complement components, α-1-acid glycoprotein 1 and 2, cysC, monocyte differentiation antigen CD14, fibrinogen and uteroglobin contribute to signaling in the immune system, inflammation, cytokine secretion, and the acute phase response. These proteins differentiated both CKD and CVD patients from HVs. However, the differences in the relative abundance of both comparisons were completely different. For example, the accumulation of α1m was only 1.4 times higher in CVD patients and up to 5.6 times higher in CKD5 patients compared to HVs (Figure 3a). In a similar situation, we observed an abundance of β2m. The level of this protein was tens of times higher in CKD patients than CVD patients. The abundance of cysC, a well-known marker of renal failure [13], was increased in the plasma of CVD patients compared to HVs (fold change of 1.53 in the ELISA). However, the accumulation of cysC in the plasma of the CKD3-4 and CKD5 patients was several times greater. The level of this protein was similar between the CVD and CKD1-2 patients (Figure 5b). The diagnostic values of β2m and cysC as markers of inflammation and kidney failure have been confirmed in multiple clinical studies [14,15,16,17]. Furthermore, other studies have postulated a significant correlation between high serum cysC levels and cardiovascular risk factors in individuals with atherosclerosis and normal renal function [18,19,20]. Classical CVD is also characterized by vascular inflammation. The question is whether inflammation is more specific to CKD or CVD. The direct comparison of the blood plasma proteomes isolated from CKD and CVD patients performed in this study, also confirmed by our previous findings [21], shows that classical CVD is related to inflammation but to a lesser extent than CKD. This finding is also confirmed by the calculated level of serum CRP, the most important biomarker of systemic inflammation.

The high concentration of circulating uremia-specific toxins also contributed to inflammation and endothelial dysfunction long before renal replacement therapy [22]. Furthermore, oxidative stress, acidosis, and the accumulation of mediators in renal failure (advanced glycation end (AGE) products, pro-inflammatory cytokines) may contribute to inflammation [9,23]. Our results showed that the plasma marker of endothelial activation vascular adhesion molecule-1 (VCAM-1) was increased in the later stages of CKD (CKD3-4 and CKD5), whereas this marker was undetectable in the plasma of the CVD, CKD1-2, and HV groups (Figure 6b). Endothelial activation as a result of oxidative stress appears to be involved in vascular damage and pathophysiology of the cardiovascular complications of CKD. The present study showed that with the development of CKD, the plasma glutathione peroxidase was significantly decreased and that this change was positively associated with eGFR (Figure 4c). In contrast, the level of PRDX2 increased in CKD patients but not in CVD patients (Figure 5a). The accumulation of superoxide dismutase was observed only in the most advanced CKD stage (Figure 6c). Glutathione peroxidase catalyzes the reduction of hydrogen peroxide and other organic hydroperoxides into water by using glutathione as the reducing agent [24]. Therefore, this enzyme protects cell membrane lipids, proteins, and DNA against oxidative stress. The level of glutathione peroxidase in CVD patients was also decreased compared to that in HVs but was similar to that in CKD1-2 patients. These results suggest that oxidative stress, especially in ESRD patients, plays a more important role in CKD-A than in classical CVD.

Vascular calcification is another risk factor that is related to progression and mortality in CKD patients [25,26,27,28]. Osteopontin and fetuin A are expressed in atherosclerotic plaques and participate in atherosclerotic calcification. Circulating osteopontin is associated with vascular calcification and arterial stiffness in coronary artery disease [29,30]. High levels of osteopontin are associated with cardiovascular risk in CKD patients [31]. In our study, osteopontin was undetectable in HVs and non-dialyzed CKD patients (Figure 6a). The accumulation of this protein only in the plasma of ESRD patients was measured by LC-MS/MS analysis. Osteopontin was also absent in CVD patients, which confirms that the vascular calcification mechanism is associated with CKD. Furthermore, these observations are confirmed by the accumulation of another protein that participates in the process of vascular calcification, fetuin A. The concentration of fetuin A was highest in HVs and gradually decreased from CKD1-2 patients, reaching its lowest value in patients with ESRD (Figure 4b). Moreover, the concentration of this protein was the highest in CVD patients. Fetuins are carrier proteins, similar to albumins, and form soluble complexes with calcium and phosphate; thus, they are carriers of insoluble calcium and are potent inhibitors of pathological calcification [32,33]. In addition to fetuin A, fetuin B is another member of the fetuin family with a similar function (Figure 4a). Fetuin B, similarly to fetuin A, is an inhibitor of basic calcium phosphate precipitation [34]. Some reports have demonstrated that fetuin A levels are inversely correlated with coronary artery calcification in hemodialysis patients [35] and diabetic patients [36]. Information on the relationship among fetuin A levels, the degree of calcification, and mortality is less clear for patients with normal renal function as well as predialysed CKD patients. To our knowledge, this is the first study showing an association between fetuin A and atherosclerosis comparing renal and non-renal conditions. To our knowledge, this is also the first report presenting an alteration in the level of fetuin B in CKD patients. The altered levels of the inhibitors of atherosclerosis-related vascular calcification, fetuin A and B as well as osteopontin, support the notion that vascular calcification is more pronounced in CKD than in CVD. Vascular calcifications may be a more specific marker for CKD-related atherosclerosis, and this phenomenon is correlated with CKD progression.

All of the aforementioned factors, including inflammation, oxidative stress, vascular calcification, and endothelial activation, lead to endothelial injury. As a result, the underlying extracellular matrix is exposed, and platelets adhere to the vessel wall, leading to leukocyte and thrombocyte activation. This vascular “microinflammation” activates the coagulation cascade, which further accelerates vessel wall damage [9,37,38]. Therefore, the connection between the coagulation process and atherosclerosis development is beyond dispute. In our previous studies, we have revealed an elevated level of fibrinogen in the plasma of CVD patients compared to HVs [21,39]. The current results confirm our previous data and demonstrate that the levels of many proteins that are involved in hemostasis are also elevated in the plasma of CVD patients. However, changes in the accumulation of these proteins are more pronounced in CKD patients. Among the 33 differential proteins related to the hemostasis process, only five proteins differentiated the HV and CVD groups. The remaining 28 proteins differentiated the HV and CKD groups according to CKD progression. Large numbers of clinical studies on atherosclerotic disease have shown a generally increased involvement of coagulation processes in CVD [40,41]. The elevated levels of fibrinogen and other proteins related to the blood coagulation process have also been found in the sera of subjects with renal insufficiency [42]. However, no studies have demonstrated alterations of the blood coagulation proteins in both diseases to highlight the differences between them. Our results suggest that despite the relationships of different non-traditional risk factors with classical CVD, similar relationships with CKD-A are not evident. We demonstrated that patients with CKD have increased oxidative stress, vascular damage, inflammation, vascular calcification, and disturbances in the blood coagulation process at higher levels than do patients with advanced classical CVD without CKD. The relative abundances of differential proteins presented in this study revealed that CVD is similar to early stages of CKD (as in the CKD1-2 group) in its relationship with non-traditional risk factors. This knowledge was obtained through the direct comparison of the blood plasma proteomes isolated from HVs and from CKD and CVD patients. In this manner, we were able to identify the differences between the relative levels of many proteins in both diseases simultaneously.

## 4. Materials and Methods

### 4.1. Subjects and Samples

Our study protocol conformed to the Ethical Guidelines of the World Medical Association Declaration of Helsinki. Before the project commenced, appropriate approval was obtained from the Bioethical Commission of the Karol Marcinkowski University of Poznan Medical Sciences, Poznan, Poland (no. 14/07; 1 April 2007). All participating individuals provided signed informed consent for inclusion before they participated in the study. The characteristics of the studied population were presented previously [21]. The study involved 150 persons divided into five equal groups. They were matched for age and gender. All of studied patients suffered from hypertension, were non-diabetic, and non-albuminuric. The majority were patients with CKD (90 persons) who were treated by the Department of Nephrology, Transplantology and Internal Medicine at Poznan University of Medical Sciences. Based on the Kidney Disease: Improving Global Outcomes [43] and the National Institute for Health and Care Excellence [44] guidelines, the examined CKD patients were divided into three groups according to their estimated GFR (eGFR). Their eGFR was calculated by the formula developed by Levey *et al.* [45]. The first group, CKD1-2, contained patients in the initial stages of CKD with eGFR = 77.04 ± 22.9 mL/min/1.73 m^2^ (mean ± SD). The second group, CKD3-4, included pre-dialyzed patients with eGFR = 19.1 ± 8.0 mL/min/1.73 m^2^. The third group, CKD5, contained end-stage renal disease (ESRD) patients with eGFR = 5.75 ± 7.1 mL/min/1.73 m^2^ who had undergone hemodialysis for 39.6 ± 9.5 months, three times per week. The CKD patients varied in the progression of atherosclerosis (significant differences in carotid intima media thickness (CIMT) were observed) and in the percentage of cardiovascular events. The CKD1-2 group primarily showed the initial clinical consequences of hypertension and/or ischemic heart disease. In the more advanced stages of CKD, the number of people with serious symptoms and consequences of CVD was greater. Fifty-nine percent of the CKD5 patients had a history of myocardial infarction or stroke. The underlying renal diseases of the patients were hypertensive nephropathy (*n* = 33), chronic glomerulonephritis (*n* = 21), chronic interstitial nephritis (*n* = 21), polycystic kidney disease (*n* = 3), and other/unknown (*n* = 12).

A fourth group (called CVD) included 30 non-diabetic patients with a history and symptoms of atherosclerotic occlusive disease who were admitted for angiography to the Department of Internal Medicine, Division of Cardiac Intensive Care in Poznan University of Medical Sciences. All of the CVD patients had at least one artery stenosis, causing at least 50% of the lumen reduction. Sixty-eight percent of CVD patients had a history of myocardial infarction or stroke. No subjects from the CVD group had any clinical symptoms of renal dysfunction (mean eGFR = 92.7 ± 21.1). A fifth group, which served as a control group, contained 30 HVs with a mean eGFR of 123.6 ± 17.6. Persons with diabetes mellitus, acute inflammatory processes, and malignant tumors either at the time of study or within the previous 10 years were excluded from the study. All of the studied subjects were tested for atherosclerosis on the basis of their medical history (history of myocardial infarction or/and ischemic stroke), systolic and diastolic blood pressure levels, their lipid metabolism parameters, and CIMT. Although patients enrolled to this study were treated by recommended groups of drugs, related to the control of blood pressure, history of cardiovascular disease and lipid profile, such as angiotensin-converting enzyme inhibitors (ACEI), non-steroidal anti-inflammatory drugs (NSAID), β-blockers, and statins, not all of them received all of these medications. Despite of the differences in the treatment between studied groups which cannot be avoided, there were no significant impacts of drugs on the obtained results. Therefore, detailed information on this issue has been omitted in this study. The peripheral blood of the persons was collected into a closed monovette system containing EDTA and was centrifuged immediately at 1000× *g* for 15 min. The obtained supernatants were then centrifuged at 16,000× *g* for 15 min at 4 °C and frozen at −80 °C. It should be emphasized that all of the analyses conducted in the CKD5 group were carried out on blood samples collected immediately before the mid-week hemodialysis session, as is usually recommended in scientific research.

### 4.2. In-Solution Trypsin Digestion

One microliter of each plasma sample without depletion was diluted with MiliQ water to a final volume of 60 μL. The protein concentration was determined using a bicinchoninic acid (BCA) (Pierce) assay. Then, 10 μg of plasma protein was reduced in the presence of 50 mM NH_4_HCO_3_ with 5.6 mM DTT for 5 min at 95 °C. Then, the sample was alkylated with 5 mM iodoacetamide for 20 min in the dark at room temperature (RT). The proteins were digested with 0.2 μg of sequencing-grade trypsin (Promega, Mannheim, Germany) overnight at 37 °C. Each plasma sample was prepared for digestion in duplicate.

### 4.3. NanoLC-MS/MS Analysis

For each run, 1.5 μg of the digested protein samples was injected onto an RP C18 precolumn (Thermo Fisher Scientific, Waltham, MA, USA) connected to a 75 μm i.d. × 25 cm RP C18 Acclaim PepMap column with a particle size of 2 μm and a pore size of 100 Å (Thermo Fisher Scientific) using a Dionex UltiMate 3000 RSLCnano System (Thermo Fisher Scientific). Every sample was injected in duplicate at random. Every 19 sample injections, the system was calibrated using Pierce LTQ ESI Positive Ion Calibration Solution (Thermo Fisher Scientific). Then, 19 freshly digested samples were injected without any break. The following LC buffers were used: buffer A (0.1% (*v*/*v*) formic acid in Milli-Q water) and buffer B (0.1% formic acid in 90% acetonitrile). The peptides were eluted from the column with a constant flow rate of 300 nL·min^−1^ with a linear gradient of buffer B from 5% to 65% over 208 min. At 208 min, the gradient increased to 90% B and was held there for 10 min. Between 218 and 230 min, the gradient returned to 5% to re-equilibrate the column for the next injection. The peptides eluted from the column were analyzed in the data-dependent MS/MS mode on a Q-Exactive Orbitrap mass spectrometer (Thermo Fisher Scientific). The instrument settings were as follows: the resolution was set to 70,000 for MS scans, and 17,500 for the MS/MS scans to increase the acquisition rate. The MS scan range was from 300 to 2000 *m*/*z*. The MS AGC target was set to 1 × 10^6^ counts, whereas the MS/MS AGC target was set to 5 × 10^4^. Dynamic exclusion was set with a duration of 20 s. The isolation window was set to 2 *m*/*z*.

### 4.4. Qualitative Analysis of Proteomic Data

After each LC-MS/MS run, the raw files were qualitatively analyzed by Proteome Discoverer (PD), version 1.4.14 (Thermo Fisher Scientific). To evaluate the quality of the performed runs, the number of peptide spectrum matches (PSMs) and the number of identified proteins were calculated. The LC-MS/MS runs with the number of PSMs below 125,000 and the number of identified proteins below 450 (with 1% FDR) were excluded from further analysis. The identification of proteins by PD was performed using the SEQUEST engine against the UniProt Complete Proteome Set of Humans (123,619 sequences) using the following parameters: a tolerance level of 10 ppm for MS and 0.05 Da for MS/MS. Trypsin was used as the digesting enzyme, and two missed cleavages were allowed. The carbamidomethylation of cysteines was set as a fixed modification, and the oxidation of methionines was allowed as a variable modification.

### 4.5. Quantitative Analysis of Proteomic Data

The raw files positively evaluated by PD were quantitatively analyzed by MaxQuant [46,47], version 1.5.1.2 (Available online: http://www.coxdocs.org website). The database search engine Andromeda was used to search the MS/MS spectra against the UniProt database, with the same parameters as for PD at ≤1% FDR. The analysis of the plasma samples was based on the label-free quantification (LFQ) intensities. The data were evaluated, and the statistics were calculated using Perseus software (version 1.4.1.3, Max Planck Institute of Biochemistry, Martinsried, Germany). The MQ data were filtered for reverse identifications (false positives), contaminants, and proteins “only identified by site”. The mean LFQ intensities as well as the standard deviation of this value were calculated for all experimental groups. The fold changes in the level of the proteins were assessed by comparing the mean LFQ intensities among all experimental groups. A protein was considered to be differentially expressed if the difference was statistically significant (*p* < 0.05), the fold change of minimum was ±1.5, it was identified with a minimum of two peptides with >99% confidence.

### 4.6. Assessment of Variability/Reproducibility

The technical and biological variabilities of each plasma sample from each experimental group were estimated by scatter plot and calculated using the Pearson correlation coefficients of the LFQ intensities in Perseus. To assess the reproducibility, the percentage overlap between the protein identification in both the technical/injection and biological replicates was calculated using PD software (Thermo Fisher Scientific, Waltham, MA, USA).

### 4.7. ELISA Validation

An ELISA was used to validate the differentially expressed proteins. The plasma protein levels were measured using a commercially available sandwich colorimetric ELISA kit (Abcam, Cambridge, UK or Elabscience, Wuhan, China) against the following proteins: α-1-microglobulin, cystatin C, β-2-microglobulin, apolipoprotein AIV, and fibrinogen α, β, and γ. All assays were prepared according to the manufacturers’ instructions. The O.D. absorbance was read at 450 nm with an Infinite 200 PRO multimode reader (Tecan, Männedorf, Switzerland).

### 4.8. Pathway and Network Analyses of Dysregulated Proteins in Plasma Samples

Only the proteins that were quantified as unique and non-redundant were used in the subsequent analyses. Proteins were considered to be differentially expressed if the difference was statistically significant (*p* < 0.05) and the fold-change minimum was ±1.5. The dysregulated proteins were chosen based on the criterion that the protein must be quantified by a minimum of two peptides with >99% confidence. Uncharacterized proteins and fragments of immunoglobulins were excluded from the analysis. The differential proteins were subjected to analysis using the Database for Annotation, Visualization, and Integrated Discovery (DAVID) (Available online: http://david.abcc.ncifcrf.gov/) [48] and Protein ANalysis THrough Evolutionary Relationships (PANTHER) (Available online: http://pantherdb.org/) [49,50] analysis tools for identifying enriched functions, signaling pathways or networks and diseases categories. *p* values and Benjamini-corrected *p*-values below 0.05 were considered significant. The pathway analysis using the DAVID tool was based on the REACTOME, KEGG pathway, and PANTHER pathway databases.

### 4.9. Statistical Analysis

The LFQ intensities derived from all of the evaluated PD samples were considered for statistical analysis. For multiple comparisons, one-way analysis of variance (ANOVA) with a Bonferroni correction for multiple testing was performed. For the comparison between two groups, *t*-tests were performed. *p* values less than 0.05 were considered to be statistically significant. Regression and correlation analyses were also performed for the obtained results. The correlations between variables were defined by the Pearson (Perseus) and Spearman (Statistica) coefficients, and *p* values less than 0.05 were considered significant. Multivariate analyses were carried out by untargeted principal component analysis (PCA). All statistical analyses were performed using the Statistica v. 10.0 software (StatSoft, Inc., Kraków, Poland) and Perseus 1.4.1.3 which is freely available from the MaxQuant website.

## Figures and Tables

**Table 1:** Pearson correlation coefficients between the LFQ (label-free quantification) intensities from the biological and technical replicates in all experimental groups. The calculations were derived from Perseus software. HV refers to healthy volunteers, CKD refers to chronic kidney disease (numbers indicate disease stages), and CVD refers to cardiovascular disease.

Experimental Group	Correlation Coefficients in Biological Replicates	Correlation Coefficients in Technical Replicates
HVs	0.9103–0.9887	0.9894–0.9967
CKD1-2	0.8711–0.9747	0.9784–0.9960
CKD3-4	0.8603–0.9774	0.9531–0.9923
CKD5	0.8391–0.9757	0.9196–0.9857
CVD	0.8110–0.9721	0.9466–0.9975

**Table 2:** Top pathways enriched in the differentially expressed proteins—results from DAVID (Database for Annotation, Visualization, and Integrated Discovery). NS: non-significant.

Pathway	Database	HV/CKD1-2	HV/CKD5	HV/CVD	Benjamini Corrected *p*-Value
		(% of Whole Proteins)	(% of Whole Proteins)	(% of Whole Proteins)	
Hemostasis	REACTOME	23.8	19.7	23.3	3.1 × 10^−6^/4.2 × 10^−7^/9.6 × 10^−6^
Complement cascade	KEGG	23.9	13.6	9.3	3.6 × 10^−11^/4.1 × 10^−8^/4.5 × 10^−2^
Blood coagulation	PANTHER	17.5	25	13.7	1.2 × 10^−1^/5.7 × 10^−12^/1.8 × 10^−7^
Inflammation mediated by chemokine and cytokine signaling pathway	PANTHER	8.3	8.3	4.8	2.5 × 10^−5^/6.4 × 10^−5^/2.4 × 10^−6^
Integrin cell surface interaction	REACTOME	15.2	12.1	–	1.1 × 10^−4^/1.5 × 10^−6^/NS
Signaling in immune system	REACTOME	12.1	19.6	–	2.8 × 10^−3^/2.5 × 10^−3^/NS
Plasminogen activation cascade	PANTHER	7.6	10.9	–	5.6 × 10^−5^/7.1 × 10^−5^/NS
Cardiac muscle contraction	KEGG	–	–	9.3	NS/NS/3.2 × 10^−2^
Cardiomyopathy	KEGG	–	–	14.6	NS/NS/2.7 × 10^−2^
Metabolism of lipids and lipoproteins	PANTHER	8.3	3.1	8.3	2.4 × 10^−4^/2.1 × 10^−3^/2.8 × 10^−5^

**Table 3:** A list of 29 proteins associated with CKD progression and consequently with the eGFR level. Eight proteins were specific only for advanced stages of CKD. The correlation coefficients were determined using the estimated glomerular filtration rate (eGFR) of the plasma samples and the LFQ intensities of proteins. The molecular functions/pathways for all proteins were defined using DAVID tools and GO annotations. The fold changes were calculated against the HV group, and the fold changes were only calculated against CKD1-2 for the two proteins (peroxiredoxin-2 and cysC). The differences identified as significant (with fold change >1.5 or <0.66 and *p* < 0.05) are in bold.

Protein	Correlation Coefficient	ANOVA	CKD1-2/HV	CKD3-4/HV	CKD5/HV	CVD/HV	Pathway/Process
Transferrin	0.750	8.6 × 10^−11^	0.88	**0.62**	**0.56**	0.94	Hemostasis
Vitronectin	0.770	1.7 × 10^−17^	0.91	0.77	**0.65**	0.99	Hemostasis
Hepatocyte growth factor activator	0.719	0.0041	0.77	**0.47**	**0.53**	0.71	Hemostasis
Glutathione peroxidase 3	0.760	3 × 10^−14^	0.81	**0.33**	**0.16**	0.8	Reactive oxygen species (ROS) detoxification
Peroxiredoxin-2	−0.7195	0.0049	–	**2.03**	**2.06**	–	ROS detoxification
Superoxide dismutase	present only in CKD5	0.0243	–	–	–	–	ROS detoxification
Fetuin A	0.730	0.0451	0.87	**0.5**	**0.46**	1.04	Calcium metabolism
Fetuin-B	0.779	6.1 × 10^−5^	0.71	**0.35**	**0.4**	0.69	Calcium metabolism
Fibrinogen α	−0.735	1.5 × 10^−13^	**1.59**	**1.76**	**1.85**	1.34	Complement and hemostasis
Fibrinogen β	−0.770	1.2 × 10^−12^	**1.59**	**1.85**	**2.05**	1.45	Complement and hemostasis
Fibrinogen γ	−0.735	3.9 × 10^−11^	**1.61**	**1.63**	**1.9**	1.19	Complement and hemostasis
β2m	−0.791	2.2 × 10^−44^	**2.46**	**8.09**	**32.04**	**1.52**	Signaling in immune system
Complement component C7	−0.797	0.0013	1.04	1.25	**1.75**	1.02	Complement and blood coagulation, immune response
Complement factor H-related protein 1	−0.706	5.5 × 10^−13^	1.49	1.43	**2.05**	1.17	Complement and blood coagulation, immune response
Coagulation factor XIII B chain	−0.720	2.4 × 10^−18^	1.2	1.22	**1.66**	1.04	Complement and blood coagulation, immune response
EGF-containing fibulin-like extracellular matrix protein 1	−0.740	8.8 × 10^−13^	**1.7**	**2.47**	**2.45**	0.88	Molecules associated with elastic fibers
Inter-α-trypsin inhibitor heavy chain H3	−0.732	7 × 10^−9^	1.08	**1.57**	**1.63**	1.4	No hits
Leucine-rich α-2-glycoprotein	−0.701	3.4 × 10^−9^	1.06	**1.82**	**2.04**	**1.57**	No hits
Peptidase inhibitor 16	−0.681	4.6 × 10^−15^	1.09	**2.89**	**3.57**	1.03	No hits
Guanylin	present only in CKD5	8.2 × 10^−13^	–	–	–	–	No hits
Protein AMBP; α1m	−0.790	5.1 × 10^−54^	**2.04**	**2.86**	**4.89**	1.4	Scavenging of heme from plasma, inflammation mediated by chemokine and cytokine signaling
Apolipoprotein C-III	−0.761	0.0003	1.33	**1.58**	**1.61**	1.02	Metabolism of lipids and lipoproteins
α-1-acid glycoprotein2	−0.706	2.2 × 10^−6^	1.26	1.27	**1.52**	1.11	Regulation and signaling in immune system
α-1-acid glycoprotein1	−0.749	3 × 10^−8^	1.32	1.47	**1.72**	1.27	Regulation and signaling in immune system
Retinol-binding protein 4	−0.770	7.3 × 10^−38^	1.35	**1.94**	**3.29**	0.91	Retinoid metabolism and transport
CysC	−0.826	8.4 × 10^−27^	–	**4.25**	**6.32**	0.85	Response to stimuli, cellular response to oxidative stress
Zinc-α-2glycoprotein	−0.716	2.6 × 10^−22^	1.1	**1.86**	**2.27**	1.39	Immune response, miscellaneous transport and binding events
Lumican	−0.769	2.7 × 10^−13^	1.07	1.38	**1.58**	1.06	Integrin cell surface interactions
β-2-glycoprotein 1	−0.813	1.4 × 10^−7^	1.2	**1.54**	**1.63**	1.2	Blood coagulation
Pigment epithelium-derived factor	−0.799	9.7 × 10^−45^	1.26	**1.61**	**2.12**	1.11	Blood coagulation
Monocyte differentiation antigen CD14	−0.771	0.0001	**2.02**	**2.85**	**3.68**	**1.55**	Immune response
Vascular cell adhesion molecule 1	present only in CKD3-4 and CKD5	0.0312	–	–	–	–	Integrin cell surface interactions, immune response
Prostaglandin-H2 d-isomerase	present only in CKD3-4 and CKD5	7.4 × 10^−2^	–	–	–	–	Synthesis of prostaglandins and thromboxanes, hemostasis
Osteopontin	present only in CKD5	4 × 10^−1^	–	–	–	–	Integrin cell surface interactions
Calreticulin	present only in CKD5	0.0479	–	–	–	–	Calcium ion binding, chaperone
CD59 glycoprotein	present only in CKD5	5.9 × 10^−11^	–	–	–	–	Regulation of complement cascade
Uteroglobin	present only in CKD5	1.6 × 10^−1^	–	–	–	–	Immune response

## Supplementary Materials

Supplementary materials can be found at http://www.mdpi.com/1422-0067/17/5/631/s1. Table S1. Complete list of differential proteins identified in plasma of HVs, CKD1-2, CKD3-4, CKD5, and CVD patients. Table provides information about the name of proteins, number of identified peptides, ANOVA and *t*-test *p* values, fold changes for all group comparisons, SwissProt accession number ,and mean LFQ intensities for all analyzed experimental group; Table S2. Twenty proteins revealed similar accumulation between CVD and CKD1-2 patients and completely different between CVD and CKD5.

## Abbreviations

CKD	chronic kidney disease
CVD	cardiovascular disease
CKD-A	CKD-related atherosclerosis
HV	healthy volunteer
GFR	glomerular filtration rate
LFQ	label-free quantification
LC-MS/MS	liquid chromatography tandem mass spectrometry
DTT	dithiothreitol
α1m	α-1-microglobulin
β2m	β-2-microglobulin
cysC	cystatin C
